# Variant Creutzfeldt-Jakob disease in UK children after 27 years of active prospective surveillance

**DOI:** 10.1136/archdischild-2025-328472

**Published:** 2025-04-02

**Authors:** Christopher Verity, Elaine Baker, Polly Maunder, Anne Marie Winstone, Suvankar Pal

**Affiliations:** 1 Paediatrics, Addenbrooke’s Hospital, Cambridge, UK; 2 Addenbrooke’s Hospital, Cambridge, UK; 3 The National Creutzfeldt-Jakob Disease Research and Surveillance Unit, Western General Hospital, Edinburgh, UK

**Keywords:** Paediatrics, Epidemiology, Neurology, Genetics

## Abstract

**Objective:**

To determine whether any children in the UK had variant Creutzfeldt-Jakob disease (vCJD).

**Design:**

This active prospective epidemiological study used the British Paediatric Surveillance Unit, asking UK paediatricians to notify all childhood cases of progressive intellectual and neurological deterioration (PIND), a group that would include all cases of vCJD. Clinical data were obtained by questionnaire or via a site visit. An independent expert group classified the cases. If vCJD was suspected, referral to the National Creutzfeldt-Jakob Disease Research and Surveillance Unit was recommended.

**Results:**

Between May 1997 and April 2024 (27 years), 5222 children were notified. There were four groups. (1) 2540 were ‘not cases’—they did not meet the case definition or there were notification errors. (2) 2367 had a known underlying diagnosis other than vCJD; the group contained more than 220 different diseases. (3) 309 had no diagnosis to explain their deterioration; there was evidence that none of these cases had vCJD. (4) There were six cases of vCJD: two males and four females. They developed symptoms between 1998 and 2000, aged 12–15 years, and the last two died in 2003. Their clinical features were similar to those of adults. Four were classified as definite vCJD and two as probable vCJD.

**Conclusions:**

This study has provided unique data about neurodegenerative diseases in UK children. There is no reliable vCJD screening test; so for 27 years, the PIND study has provided reassurance that childhood vCJD cases were not missed. New vCJD cases with the methionine/valine genotype could appear.

**WHAT IS ALREADY KNOWN ON THIS TOPIC**

- After variant Creutzfeldt-Jakob disease (vCJD) was reported in adults in 1996, surveillance in children became a public health priority.

**WHAT THIS STUDY ADDS**

- Six children with vCJD were identified. No cases have been detected in the UK since the last two died in 2003.

**HOW THIS STUDY MIGHT AFFECT RESEARCH PRACTICE OR POLICY**

- The progressive intellectual and neurological deterioration study has provided the only means of screening UK children for vCJD. It is not clear how this screening will be carried out now that the study has ended.

## Introduction

 The first report of bovine spongiform encephalopathy (BSE) in British cattle was published in 1987^1^; it was subsequently suggested that the disease was caused by feed contaminated with a scrapie-like agent.^2^ Amid increasing concern about the BSE epidemic, the National Creutzfeldt-Jakob Disease Surveillance Unit (NCJDSU) was established in Edinburgh in 1990. The Edinburgh unit asked neurologists and neuropathologists to notify suspected cases of adult Creutzfeldt-Jakob disease (CJD). Most of the reported cases were of sporadic CJD (a previously recognised rare disorder of the central nervous system), but in 1996, Will and colleagues reported 10 cases of a new variant of CJD (now referred to as variant CJD (vCJD)), with an earlier onset than sporadic CJD, a different clinical presentation and a new neuropathological phenotype. Exposure to the BSE agent was suggested as the cause.^3^ Within months, a ban on the export of British beef to the European Union was in place.

There was a call for further epidemiological surveillance to investigate this important public health issue.^4^ The newly described vCJD occurred in a younger age group than sporadic CJD—the youngest case described in 1996 developed symptoms at 16 years of age.^3^ This finding raised concern that children might develop the disease. Two major difficulties in planning surveillance for vCJD among children were that the clinical presentation in young children might be different from that in adults and that there are many rare neurodegenerative diseases in childhood.

In 1996, a decision was made to carry out surveillance for children with ‘progressive intellectual and neurological deterioration’ (PIND), defining a group of children that would include any with vCJD. The PIND study used the active national surveillance mechanism of the British Paediatric Surveillance Unit (BPSU), established in 1986.^5^ The study was carried out in close collaboration with the NCJDSU (later Research and Surveillance Unit (NCJDRSU)).

When the study started, an orange surveillance card was being posted by the BPSU to all UK consultant paediatricians. This was replaced by an online system, with up to a dozen rare disorders simultaneously under surveillance. Paediatricians report cases seen in the previous month (or indicate that they have seen none of the conditions under surveillance). The BPSU informs the investigators about paediatricians who have notified cases, and the investigators obtain clinical data from the notifiers. There has been an excellent response to the BPSU system. In 1997, more than 2000 cards were posted each month, and over 90% were returned. Between 2016 and 2020 inclusive, the BPSU annual response rates varied between 88% and 94%.^6^

Since May 1997, the PIND study has obtained information from paediatricians who notified cases using the case definition for PIND (see box 1). The clinical data were obtained by questionnaire, telephone interview with the child’s paediatrician or via a visit to extract data from the hospital notes. Name and date of birth were needed so that the PIND study could gather follow-up information from clinicians involved in the child’s care and obtain investigation results from laboratories, some outside the UK. Classification of ethnicity was that used by the Communicable Disease Surveillance Centre, Colindale, London, UK, when the PIND study commenced in 1997. The categories were White, Black Caribbean, Black African, Black other, Indian, Pakistani, Bangladeshi, Chinese, Other. Patient-identifiable information was kept secure—the name was removed before clinical details were held on a password-protected computer database. Records that were no longer needed were anonymised for storage.

Box 1 **Case definition of progressive intellectual and neurological deterioration**

**Excluding**

Static intellectual loss—for example, after encephalitis, head injury or near drowning.

**Including**

Neurological disorders with specific diagnoses and meeting the case definition. Metabolic disorders leading to neurological deterioration; seizure disorders if associated with progressive deterioration; known presymptomatic cases of diagnosed neurodegenerative conditions. Reporting is restricted to cases seen in the previous month but includes those whose disorder began earlier (ie, including ‘old cases’ of children in follow-up if seen in that month).

A PIND study expert group provided independent opinion and advice, meeting every 3 months, initially in the Royal College of Paediatrics and Child Health, but with the arrival of COVID-19, remotely using Microsoft Teams. The group included specialists in paediatric neurology, metabolic disease and neurogenetics. For the meetings to be quorate, two paediatric specialists and a representative from the NCJDRSU had to be present. The PIND study team provided anonymised summaries of the clinical details and investigations for each notified PIND child. Cases were discussed by the group to identify any that fulfilled the criteria for definite or probable vCJD (see box 2) and to categorise them as described in detail in the Results section. Cases still under investigation were discussed again when more information was available. After the final confirmation of a diagnosis, the notifying paediatrician was informed, and follow-up by the study ceased. The study also stopped follow-up of undiagnosed cases when the patient died or when the clinicians who were managing the case had no plans for further investigations.

Box 2 **Diagnostic criteria for variant CJD**

**I**

- Progressive neuropsychiatric disorder.
- Duration of illness >6 months.
- Routine investigations do not suggest an alternative diagnosis.
- No history of potential iatrogenic exposure.

**II**

- Early psychiatric symptoms.*
- Persistent painful sensory symptoms.†
- Ataxia.
- Myoclonus, chorea or dystonia.
- Dementia.

**III**

- Electroencephalogram does not show the typical appearance of sporadic CJD‡ (or no electroencephalogram was done).
- Bilateral pulvinar high signal on MRI.

**Definite:** IA (progressive neuropsychiatric disorder) and neuropathological confirmation of variant CJD.§

**Probable:** I and 4/5 of II and IIIA and IIIB.

**Possible:** I and 4/5 of II and IIIA.

*Depression, anxiety, apathy, withdrawal and delusions.†This includes both frank pain, unpleasant dysaesthesia or both.‡Generalised triphasic complexes at about 1/s.§Spongiform change and extensive prion protein deposition with florid plaques throughout the cerebrum and cerebellum.CJD: Creutzfeldt-Jakob disease.

The PIND team did not become involved with patients or their families. Paediatricians who notified children had confidential feedback from the PIND expert group, but the notifying paediatricians were responsible for clinical management.

It was agreed that cases with features suggestive of vCJD would be discussed with the local paediatrician, recommending referral to the NCJDRSU after obtaining consent from the relatives. The NCJDRSU then made contact with the family to arrange the appropriate investigations.

## Results

### Notification rates

The study commenced in May 1997. After an initially large number of notifications of prevalent cases in the first 20 months of the study, the number of notified cases of suspected PIND settled over the period 1999–2018 inclusive to between 146 and 230 per year—this notification rate continued to be relatively constant. In 2019, the lifetime risk at birth of having a disease causing PIND was calculated using the 1703 diagnosed cases meeting the PIND criteria who were notified between the beginning of 1999 and the end of 2018. During that period, there were 14 935 076 live births in the UK,^7^ and so the lifetime risk of having a disease causing PIND was 0.1 per 1000 live births.^8^

Despite the disruption caused by the COVID-19 pandemic, the PIND study notification rate remained quite steady. From 2019, until the study stopped active surveillance at the end of 2023, it was between 121 and 171 per year. After that, six cases of suspected PIND were notified, the last in April 2024.

### Final classification of cases notified to the study

**Figure 1:**
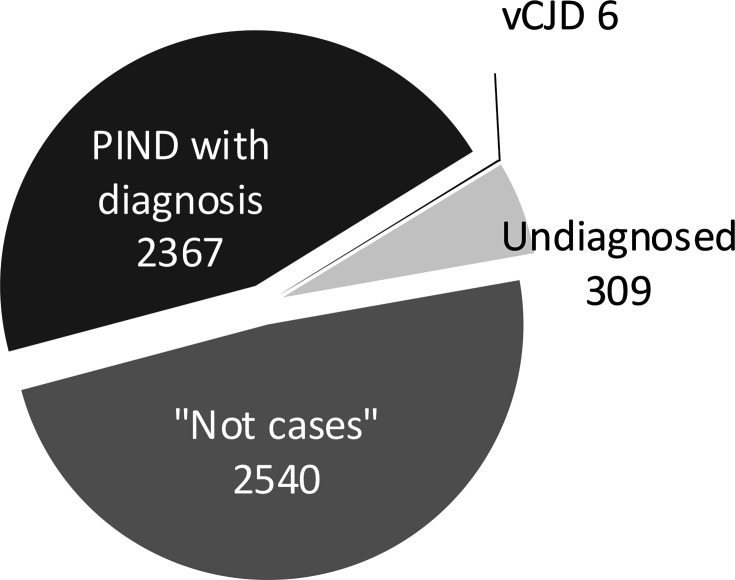
The final classification of the 5222 cases reported to the PIND study between May 1997 and April 2024. PIND, progressive intellectual and neurological deterioration; vCJD, variant Creutzfeldt-Jakob disease.

By April 2024, when the study had been running for 27 years, 5222 children had been notified. After follow-up, details and investigation results had been obtained, the cases were classified into the groups shown in figure 1. Those in the largest group (n=2540) are described as ‘not cases’. This group included those who did not meet the PIND case definition, multiple notifications, reporting errors and cases where a paediatrician notified a child but then could not supply further information. It was reassuring that the ‘not cases’ made up a large group, suggesting active involvement by UK paediatricians. It was better to be notified about possible cases and then exclude those not meeting the PIND study criteria than not to know about them.

The next largest group, PIND with diagnosis (n=2367) was made up of children who met the case definition for PIND and had a known underlying diagnosis to explain their deterioration other than vCJD. All these cases were discussed by the expert group. The cases were heterogeneous, comprising more than 220 different diseases. The number increased with time, partly because advances in molecular genetics resulted in more specific diagnoses being made. The distribution of diseases causing PIND varied with age and ethnic group, as published in a previous analysis of four age groups in White and Pakistani children.^8^

There were 309 undiagnosed children who met the criteria for PIND but had no known diagnosis to explain their deterioration. They were reviewed repeatedly by the expert group. They had usually been thoroughly investigated; for example, 90% (278/309) underwent MRI brain scans—there were no reports of the high signal in the pulvinar of the thalamus described in vCJD. No undiagnosed cases had the clinical features described in older patients with vCJD. As vCJD in children might be different from adults, the expert group looked for a new childhood phenotype of vCJD. There was no evidence of this in the undiagnosed group. A high proportion had consanguineous parents—130 (48%) of the 272 cases where the information was available—suggesting that many of them had currently unrecognised inherited neurodegenerative diseases. Only a small proportion of the undiagnosed cases had either antemortem or postmortem neuropathology performed (see below). This is a concern because a neuropathological study, with prion protein staining of neural tissue, is the only way to confirm or exclude vCJD.

### Neuropathology in undiagnosed children

In 6 of the 309 undiagnosed children, a brain biopsy was performed during life. In one, the neuropathology was reviewed by the NCJDRSU—staining for abnormal prion protein was negative. None of the other biopsies had prion protein staining. In 212 of the 309 cases, the child’s death had been reported and confirmed via the National Health Service England Digital Spine.^9^ In 172 of the 212 children reported dead, it was known whether or not postmortem examinations had been carried out. Twelve of the 172 had postmortem studies: one had a liver biopsy, another had liver and muscle biopsies, and there were 10 autopsies. A neuropathological study was carried out in seven. In one of the seven, the brain tissue was sent to the NCJDRSU, where staining for abnormal prion protein was negative. In another case, the neuropathology was reviewed by the NCJDRSU and was not that of vCJD. Thus, prion protein staining of brain tissue was carried out in just 2 of the 309 undiagnosed children—in a brain biopsy during life and in brain tissue obtained at autopsy. The numbers that had postmortem neuropathology in different ethnic groups were: White 4, Pakistani 2 and Indian 1

### vCJD cases

In 1999, three young people were notified who had developed symptoms before the age of 16 years and had vCJD: these have been previously reported.^10^ Later, three more cases were notified, the last one developing symptoms in 2000. All six cases were investigated by the NCJDRSU. The clinical details are summarised in table 1.

**Table 1:** Characteristics of the six children with vCJD notified to the study

Sex	Male	Female	Female	Male	Female	Female
Year of birth	1982	1985	1984	1983	1987	1986
Approximate date of onset (month/year)	07/1998	11/1998	05/1999	10/1999	12/2000	11/2000
Age at onset	15 years	12 years	14 years	15 years	13 years	13 years
Psychiatric or behavioural symptoms	Yes	Yes	Yes	Yes	Yes	Yes
Cognitive deterioration	Yes	Yes	Yes	Yes	Yes	Yes
Neurological symptoms	Yes	Yes	Yes	Yes	Yes	Yes
Abnormal neurological signs	Yes	Yes	Yes	Yes	Yes	Yes
MRI: high signal in pulvinar of thalamus	Yes	Yes	Yes	Yes	Yes	Yes
Cerebrospinal fluid14–33*	Negative	Positive	Negative	Negative	Positive	Negative
Mutations ofprion protein gene	MM	MM	MM	MM	Not done	Not done
Year of death (month/year)	02/2000	11/2000	01/2000	02/2001	01/2003	04/2003
Approximate onset-death interval	1 year 7 months	2 years 0 months	8 months	1 year 4 months	2 years 1 month	2 years 5 months
Postmortem showing typical neuropathology	Yes	Yes	Yes	Yes	No post mortem	No post mortem
Diagnosis	Definite vCJD	Definite vCJD	Definite vCJD	Definite vCJD	Probable vCJD	Probable vCJD

* Cerebrospinal fluid 14-3-3 protein useful in diagnosing sporadic CJD but less sensitive and specific in vCJD.

MM, methionine homozygous; vCJD, variant Creutzfeldt-Jakob disease.

There were two males and four females, born between 1982 and 1987. In all six, the ethnicity was White. Two developed their symptoms in 1998, two in 1999 and two in 2000. Their age at the onset of symptoms ranged from 12 years to 15 years; it was difficult to be exact about the timing because the onset was gradual. The interval between developing symptoms and being seen by the paediatrician who notified the case varied between 6 to 8 weeks and 3 to 4 months. The earliest problems were behavioural or psychiatric, including anxiety, agitation, depression, disinhibited behaviour, temper outbursts and aggressive behaviour. These appeared at about the same time as a deterioration in schoolwork. Neurological symptoms soon followed; in general, they were present by the time the child was first seen by the notifier: poor coordination, an unsteady gait, difficulties with speech and swallowing, sometimes involuntary movements and in one case bilateral leg pains. Abnormal neurological signs included ataxia, dysarthria, abnormal movements (chorea and dystonia), myoclonic jerks, variable tone in the limbs, in one case loss of vibration sense from the waist down and in another tics.

In all cases, brain MRI scans showed a high signal in the pulvinar of the thalamus. Four cases were methionine homozygous at codon 129 of the prion protein gene; in two cases, there was no evidence of molecular genetic testing. Sadly, all the cases died: three in 2000, one in 2001 and two in 2003. The interval between the onset of symptoms and death varied from 8 months to 2 years and 5 months. Four had postmortem neuropathology that showed characteristic spongiform change, florid amyloid plaques and positive immunohistochemical staining for abnormal prion protein; they were diagnosed as definite vCJD. The other two did not have postmortem studies and were diagnosed with probable vCJD.

## Discussion

The PIND study carried out prospective epidemiological surveillance of neurodegenerative diseases in UK children for 27 years, starting in May 1997. It provided reassurance that no vCJD cases in children occurred after 2003 and gathered unique data about more than 220 neurodegenerative diseases in the 2367 cases with an underlying diagnosis.

### Possible limitations of the study

Notified patients were not followed up by the study after a diagnosis was made. In undiagnosed cases, follow-up ceased when no further investigations were planned or when the child died, although occasionally there was subsequent information that a diagnosis had been made. However, the study did not provide systematic long-term follow-up.

Another possible limitation was that vCJD might have been hidden among the 309 undiagnosed cases—see previous PIND study publications.^11 12^ These children had usually been thoroughly investigated, and none had the clinical features described in older patients with vCJD. Childhood vCJD might have different clinical features from those in adults, but there was no evidence of a new phenotype of vCJD in children. The high level of consanguinity in the parents of undiagnosed children (48%) suggested that many of those children had currently unrecognised inherited neurodegenerative diseases. The PIND study expert group provided an independent evaluation of the undiagnosed children. These experts demonstrated great commitment; there were over 100 meetings of the expert group in 27 years; none were cancelled because they were not quorate.

In 2019, the PIND study reported that the diagnosis was made without brain biopsy or postmortem neuropathology in 99% of the 1819 diagnosed children with PIND.^12^ Only 24 of the children in the diagnosed group were known to have had postmortem examinations. However, these did help to make the diagnosis in 15 cases (four cases of Alpers syndrome, two cases of pontocerebellar hypoplasia, two cases of neuroaxonal dystrophy and single cases of Krabbe disease, GM1 gangliosidosis, Alexander disease, Rasmussen encephalitis, Niemann-Pick type C, astrocytoma and Leigh syndrome). It is, therefore, a concern that only 13 of the 309 undiagnosed PIND children had either antemortem brain biopsies (six cases) or postmortem neuropathology (seven cases), and only two had prion protein staining of the brain sample (these numbers differ from those in the previous paper^12^ because some in that undiagnosed group were diagnosed later). The only means of confirming or excluding vCJD is by prion protein staining of neural tissue, so it was not possible to be absolutely certain that cases of vCJD were not hidden in the undiagnosed group. However, as childhood neurodegenerative diseases are usually diagnosed by investigations during life and the expert group reviewed the investigations in the undiagnosed group in detail, there is reassurance that vCJD cases were not missed.

Another possible limitation was underreporting of PIND cases. In 2004, the PIND study reported on the different numbers of PIND cases (by residence) in the 126 Public Health Laboratory Service UK health districts. Only six health districts had not reported any PIND cases, suggesting good coverage by the study.^13^

### vCJD in children

The study identified six children with vCJD. The first developed symptoms in 1998; the last two developed symptoms in 2000, and both died in 2003. No cases of vCJD in UK children have been identified since then.

In 2002, a paper described the first 100 cases of vCJD, noting that within 4 months of onset, a high proportion of patients have a combination of psychiatric and neurological features that suggest vCJD.^14^ The clinical features of the vCJD children identified by the PIND study were very similar to those described in adults.

In 2011, the PIND data were combined with those of the NCJDRSU to find no evidence of vertical transmission from mother to child.^15^ There remains the possibility that vCJD could be transmitted from adults to children via blood products or during dental or surgical procedures. It is reassuring that no cases of vCJD in UK children have been identified since the last two presented in 2000.

### Implications for the future

Until 2016, all cases (including children) of vCJD in the UK who were tested were methionine homozygous at codon 129 of the prion protein gene on the short arm of chromosome 20. However, the last adult case of vCJD in the UK, who died in 2016,^16^ was methionine/valine heterozygous (MV). Codon 129 polymorphisms in the normal UK population are as follows: methionine homozygous 42%, valine homozygous 11% and MV 47%.^17^ There is the possibility of a resurgence of vCJD cases with the MV genotype in the future.

It seems imperative that surveillance for vCJD continues. In the absence of a reliable screening test for vCJD, the PIND study has been the only means of searching for vCJD cases in children. It has provided reasonable certainty that vCJD cases have not been missed among the many rare neurodegenerative diseases of childhood. It is not clear how surveillance can be reliably performed in children now that the PIND study has ceased. The NCJDRSU undertakes surveillance of adults but does not have the capacity to screen UK children for vCJD.

## Supplementary material

10.1136/archdischild-2025-328472 online supplemental file 1

## Data Availability

Data are available upon reasonable request.
