# Correction: *Variant Creutzfeldt-Jakob disease in UK children after 27 years of active prospective surveillance*

**DOI:** 10.1136/archdischild-2025-328472corr1

**Published:** 2025-05-20

Verity C, Baker E, Maunder P*, et al* Variant Creutzfeldt-Jakob disease in UK children after 27 years of active prospective surveillance. *Arch Dis Child* 2025;110:450–4. doi: 10.1136/archdischild-2025–3 28 472

The authors have noticed an error in the paragraph headed ‘Neuropathology in undiagnosed children’. It reads: ‘In 212 of the 309 cases, the child’s death had been reported and confirmed via the National Health Service England Digital Spine. In 172 of the 212 children reported dead, it was known whether or not postmortem examinations had been carried out. Twelve of the 172 had postmortem studies: one had a liver biopsy, another had liver and muscle biopsies, and there were 10 autopsies.’

It should read: ‘In 204 of the 309 cases, the child’s death had been reported and confirmed via the National Health Service England Digital Spine. In 114 of the 204 children reported dead, it was known whether or not postmortem studies had been carried out. Twelve of the 114 had postmortem studies: one had a liver biopsy, another had liver and muscle biopsies, and there were 10 autopsies.’

