# Supplementary material for: Variant Creutzfeldt-Jakob disease in UK children after 27 years of active prospective surveillance
Source: Arch Dis Child. 2025 Apr 2;110(6):e328472. doi: 10.1136/archdischild-2025-328472 (PMC12171403; doi:10.1136/archdischild-2025-328472)

Variant Creutzfeldt-Jakob disease in UK children after 27 years of active prospective surveillance.

PIND = progressive intellectual and neurological deterioration.

The PIND Study used the British Paediatric Surveillance Unit, asking UK paediatricians to report all cases of PIND in children, from 1997 onwards.

Objective: to identify any UK cases of variant CJD (vCJD) in childhood.
The added extra: unique data about neurodegenerative diseases.

Results: 6 cases of vCJD were identified, two males and four females, who developed symptoms between 12 and 15 years of age. The last two died in 2003. No vCJD cases have been found in UK children since then.

2367 children had an underlying diagnosis other than vCJD to explain their deterioration. There were more than 220 different diseases in this group.
There was no evidence of vCJD in the 309 children without an underlying diagnosis.

Conclusion: the PIND Study has been the only way to demonstrate that there have no cases of vCJD in UK children since 2003. The last UK adult with vCJD died in 2016, but more cases could appear.

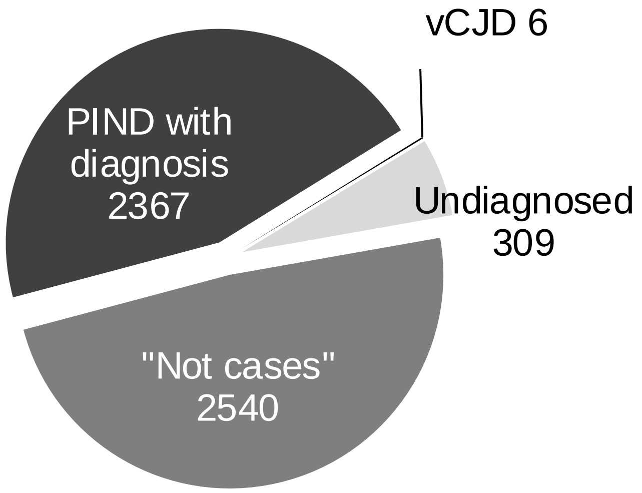

Supplement: online supplemental file 1 [file archdischild-110-6-s001.pdf]
